# Submucosal saline injection-assisted endoscopic ultrasonography for accurate layer identification of a rectal subepithelial lesion

**DOI:** 10.1055/a-2695-4195

**Published:** 2025-09-18

**Authors:** Elena De Cristofaro, Jean Grimaldi, Jérôme Rivory, Pierre Lafeuille, Florian Rostain, Alexandru Lupu, Mathieu Pioche

**Affiliations:** 1 Gastroenterology Unit, Department of Systems Medicine, University of Rome Tor Vergata, Rome, Italy; 2 Gastroenterology and Endoscopy Unit, Edouard Herriot Hospital, Hospices Civils de Lyon, Lyon, France

Endoscopic ultrasound (EUS) is a recommended technique for the evaluation of subepithelial lesions (SELs), particularly in determining their layer of origin, ranging from the mucosa to the muscularis propria 1 . However, distinguishing between the submucosal and muscular layers can sometimes be challenging, especially in the rectum where the anatomical planes are closely apposed.

Recent studies have reported that submucosal saline injection before or during EUS can improve staging accuracy in gastric and esophageal lesions 2 3 . However, evidence supporting this approach remains limited. When feasible, endoscopic resection allows for complete removal of the lesion for a definitive diagnosis, thanks to direct access to the tissue 4 .

We report a case in which submucosal saline injection was used during EUS to enhance anatomical delineation and clarify the origin of a suspected rectal SEL.

A 71-year-old man was referred for resection of a small suspected SEL located in the distal rectum. Preliminary EUS revealed a hyperechoic lesion, with an uncertain origin, either from the submucosa or the muscularis propria ( Fig. 1 **a** ).

**Fig. 1:**
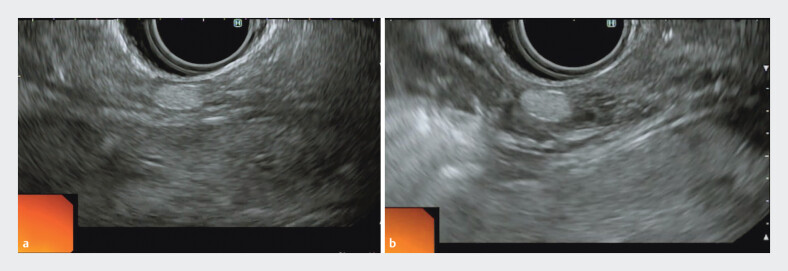
Endoscopic ultrasonography of a subepithelial lesion. **a** Before submucosal saline injection. **b** After submucosal saline injection.

A submucosal saline injection was performed circumferentially around the lesion using a 25-G needle, followed by repeat EUS. After injection, the lesion was clearly separated from the muscularis propria, confirming its origin in the submucosal layer ( Fig. 1 **b** , Video 1 ).

Injection-assisted endoscopic ultrasonography for accurate layer identification.Video 1

Nonetheless, an endoscopic intermuscular dissection was performed to ensure complete resection of the full wall and avoid an R1 vertical margin. Histopathological examination revealed a submucosal lipoma with R0 deep resection and no contact with the muscularis propria.

This case illustrates the potential utility of submucosal saline injection during EUS to improve layer discrimination when standard imaging is inconclusive. This approach may be particularly useful in preoperative decision making, especially in distinguishing lesions requiring endoscopic submucosal dissection from those better suited for other techniques such as full-thickness or endoscopic intermuscular dissection. It may also aid in the local staging of early rectal cancers where accurate identification of layer invasion is critical.

Endoscopy_UCTN_Code_TTT_1AS_2AZ

## Notes

Endoscopy E-Videos https://eref.thieme.de/e-videos
*E-Videos* is an open access online section of the journal *Endoscopy* , reporting on interesting cases and new techniques in gastroenterological endoscopy. All papers include a high-quality video and are published with a Creative Commons CC-BY license. Endoscopy E-Videos qualify for HINARI discounts and waivers and eligibility is automatically checked during the submission process. We grant 100% waivers to articles whose corresponding authors are based in Group A countries and 50% waivers to those who are based in Group B countries as classified by Research4Life (see: https://www.research4life.org/access/eligibility/ ). This section has its own submission website at https://mc.manuscriptcentral.com/e-videos .
